# Value of high‐resolution full‐field optical coherence tomography and dynamic cell imaging for one‐stop rapid diagnosis breast clinic

**DOI:** 10.1002/cam4.6560

**Published:** 2023-09-29

**Authors:** Alexis Simon, Yasmina Badachi, Jacques Ropers, Isaura Laurent, Lida Dong, Elisabeth Da Maia, Agnès Bourcier, Geoffroy Canlorbe, Catherine Uzan

**Affiliations:** ^1^ Department of Radiology, Pitié‐Salpêtrière Hospital Assistance Publique‐Hôpitaux de Paris (AP‐HP) Paris France; ^2^ Clinical Research Unit, Pitié‐Salpêtrière Hospital Assistance Publique‐Hôpitaux de Paris (AP‐HP) Paris France; ^3^ Department of Pathology, Pitié‐Salpêtrière Hospital Assistance Publique‐Hôpitaux de Paris (AP‐HP) Paris France; ^4^ Department of Gynaecological and Breast Surgery and Oncology Assistance Publique des Hôpitaux de Paris (AP‐HP) Paris France; ^5^ Centre de Recherche Saint‐Antoine (CRSA), INSERM UMR_S_938, Cancer Biology and Therapeutics Sorbonne University Paris France; ^6^ Institut Universitaire de Cancérologie (IUC) Sorbonne University Paris France

**Keywords:** breast biopsy, breast cancer, full‐field optical coherence tomography, one‐stop breast clinic diagnosis

## Abstract

**Background:**

Full‐field optical coherence tomography combined with dynamic cell imaging (D‐FFOCT) is a new, simple‐to‐use, nondestructive, quick technique that can provide sufficient spatial resolution to mimic histopathological analysis. The objective of this study was to evaluate diagnostic performance of D‐FFOCT for one‐stop rapid diagnosis breast clinic.

**Methods:**

Dynamic full‐field optical coherence tomography was applied to fresh, untreated breast and nodes biopsies. Four different readers (senior and junior radiologist, surgeon, and pathologist) analyzed the samples without knowing final histological diagnosis or American College of Radiology classification. The results were compared to conventional processing and staining (hematoxylin–eosin).

**Results:**

A total of 217 biopsies were performed on 152 patients. There were 144 breast biopsies and 61 lymph nodes with 101 infiltrative cancers (49.27%), 99 benign lesions (48.29%), 3 ductal in situ carcinoma (1.46%), and 2 atypias (0.98%). The diagnostic performance results were as follow: sensitivity: 77% [0.7;0.82], specificity: 64% [0.58;0.71], PPV: 74% [0.68;0.78], and NPV: 75% [0.72;0.78]. A large image atlas was created as well as a diagnosis algorithm from the readers' experience.

**Conclusion:**

With 74% PPV and 75% NPV, D‐FFOCT is not yet ready to be used in clinical practice to identify breast cancer. This is mainly explained by the lack of experience and knowledge of this new technic by the four lectors. By training with the diagnosis algorithm and the image atlas, radiologists could have better outcomes allowing quick detection of breast cancer and lymph node involvement. Deep learning could also be used, and further investigation will follow.

## 1 INTRODUCTION

Breast cancer is a highly incident disease. According to the World Health Organization, 2.3 million women were diagnosed with breast cancer in 2020, with 685,000 deaths worldwide.^1^ The concept of a care pathway and its application in breast cancer is crucial, but it can only be initiated following a cancer diagnosis. As a result, initiation of breast cancer care pathway can sometimes be prolonged, and it was further delayed during the COVID‐19 epidemic, highlighting system's deficiencies.^2^, ^3^

Several biopsies and histological preparations may be required for a complete diagnosis, which will technically take 48–72 h but may take weeks in daily practice. Indeed, histological diagnoses frequently take longer than expected due to a lack of resources or poor‐quality samples that necessitate redoing the entire process. Cytology enables immediate diagnosis but requires the presence of a pathologist in the sampling room, which is difficult to apply widely.^4^, ^5^

The use of a tool that would allow an immediate assessment of biopsied tissue and a potential first diagnosis without mobilizing a pathologist could thus shorten the time to initiation of care pathway, decrease patients' anxiety in anticipation of the diagnosis, and avoid delay in treatment initiation.

Full‐field optical coherence tomography (FF‐OCT) is a nondestructive imaging technique that uses light interference to reveal subcellular metabolic contrast in fresh ex vivo tissues. Dynamic cell imaging (DCI) is a complementary modality to FF‐OCT. Dynamic‐FF‐OCT (D‐FFOCT) uses intracellular cell dynamics to add a new contrast based on cell motility, metabolism,^6^ and can also reveal cell mitotic state.^7^ When combined with static FF‐OCT, the 3D structure, as well as cell distribution and shape, are recovered, providing a view of the sample similar to standard hematoxylin and eosin (H&E) histology.^8^ The contrast in the FF‐OCT images is generated by intrinsic tissue scattering properties, meaning that no tissue staining or preparation is required.^9^, ^10^ This new technique provides high‐resolution images of samples with a contrast similar to H&E histology, but without any tissue preparation nor alteration.

It is an appealing technique because it is simple‐to‐use, nondestructive, quick, and provides sufficient spatial resolution to mimic traditional histopathological analysis.

Several teams have already used FF‐OCT technology in a variety of cancers, including brain tumors, pancreatic, endometrial, lung, gut, prostate, and even breast cancer, with promising results, by pathologist readers. In breast cancer notably, Ossayag et al. obtained a 90% sensitivity and 75% specificity in detecting cancer on surgical specimens.^10^, ^11^, ^12^, ^13^, ^14^

However, to date, D‐FFOCT has not been tested in daily practice to make an immediate breast cancer and lymph node involvement diagnosis by non‐pathologist readers.

## 2 MATERIALS AND METHODS

### 2.1 Light‐CT system

The optical scanner (Light‐CT; LLTech) used for FF‐OCT and DCI is a Linnik interferometer with incoherent illumination, where one arm contains the sample to image and the other one a reference mirror. Light‐CT directly captures “en face” images on megapixel cameras at high lateral resolution (down to 1 μm) by using medium‐to‐large aperture microscope objectives and high axial resolution (1 mm) thanks to its use of a white light source. Optical scanner can then image unprocessed tissue samples down to a few hundred micrometers below the specimen surface (depending on tissue type).^6^ The acquisition relies on scanning units of 1.24 mm × 1.24 mm, which are thereafter compiled into a single image for a larger field of view. FF‐OCT image processing requires on average 2 s per unit, whereas DCI images needs 11 s per unit. To scan an entire sample, between 20 and 50 units are needed, depending on the shape and the length of the sample. FF‐OCT and DCI images are then created and directly transmit to the computer to be analyzed.

Scale bars have been added to the figures based on the predefined 1.24‐mm scanning windows.

### 2.2 Image processing and patient inclusion

After organized breast screening or self‐palpation of a breast nodule, patients' lesions were classified using the Birads classification of the American College of Radiology (ACR).^15^ ACR3–5 patients were referred for diagnosis biopsy with lymph node biopsy if there were suspicious on US. From April 28, 2020, to July 28, 2021, patients referred to Pitié Salpêtrière hospital for breast or lymph node biopsy were prospectively included. Initially, we decided to include only ACR4 and 5 biopsy‐inducing lesions. However, in our center, patients with ACR3 lesion and personal history of breast cancer were addressed for biopsy. As the aim of the study was to reflect daily practice, ACR3 lesions were included when they warranted biopsy.

The radiologist performed breast +/− lymph node micro‐biopsy. For each biopsy, one to three samples were taken depending on the material needed for standard pathology analysis. Each sample was preserved in physiological serum before imaging, then placed in the center of a cylinder slightly compressed with a thin transparent slide. The cylinder was introduced in the scanner for analysis. The samples had to be analyzed by DCI within 30 min at room temperature to keep the tissue alive in order to preserve the metabolic properties of the cells. Alternatively, it could also be stored at +4°C for a maximum of 4 h.

Two images by sample, FF‐OCT and DCI, were then scanned by the Light‐CT System at a depth of 30 μm. It took a maximum of 10 min to scan one sample by DCI and 2 min for FFOCT. Hence, for one biopsy with three samples, it could take up to 36 minutes to complete the scanning process. The preparation of the samples took on average 2 min for each. The biopsies were then sent in a formalin solution to be processed and analyzed using standard pathology techniques.

A junior radiologist, a senior radiologist, a senior pathologist, and a senior breast surgeon independently analyzed the images without knowing the final diagnosis, the Birads classification, nor the results of the other readers. Lectors analyzed, in the same order, the images obtained in DCI and FF‐OCT for each sample^1^, ^2^, ^3^ of the biopsied lesion. Then, they classified the lesions as: cancer, no cancer, or unknown. In situ cancer was classified as cancer in the analysis, and atypias were excluded from the analysis. None of the readers had been specifically trained to interpret those images. At the time of the readings, the diagnoses had already been made using standard pathology by an independent pathologist who was not participating to the study. Following the blind reading of each image, the four readers could consult the final diagnosis made using standard techniques, providing some opportunity to progressively improve their ability to interpret D‐FFOCT images. The readers did not communicate during the process but made notes of their observations and classic mistakes. After the reading process and by gathering their impressions, the readers were able to analyze together their errors, the classic pitfalls, and the typical images of cancer or benign specimens. Thus, they were able to define a reading method by creating a diagnostic algorithm. Relevant epidemiological data at diagnosis of breast cancer were collected for each patient included. Histological and molecular data of the tumors and histological types of each lesion were also collected after standard pathology.

In accordance with French law, all patients were informed about the study objectives and methods and did not oppose to the use of their personal data and biopsy samples. On February 4, 2020, the Institutional Review Board, (IRB00003888, Inserm Ethics Evaluation Committee CEEI) approved this study. The study was registered in ClinicalTrials.gov on March 3, 2020, under the identifier NCT04292821.

### 2.3 Statistical analysis

The primary objective of the study was to demonstrate that the positive predictive value of the D‐FFOCT imaging when assessed by the senior radiologist would be significantly greater than 70%, based on the lower bound of a 95% Clopper‐Pearson confidence interval.

The number of biopsies required for the study was estimated via statistical simulations assuming a cancer prevalence of 40%, a true sensitivity of 80%, and true specificity of 90%. Based on these assumptions, we estimated that 204 biopsies would provide 80% statistical power. GEE and mixed logistic models were used to ascertain the association of potential predictors with sensitivity, specificity, and positive and negative predictive values, accounting for the correlation arising from multiple readings of the same images. All tests were performed at the 5% level of confidence.

## 3 RESULTS

### 3.1 Population and technical feasibility

From May 28, 2020, to July 28, 2021, 217 biopsies were performed on a total of 152 prospectively included patients referred for suspicion of breast cancer or lymph node involvement. A total of 420 samples were scanned for the 217 biopsies performed (1.9 samples per biopsy depending on the lesion). For a biopsy with two samples, it took an average of 20 min for the entire procedure (preparation of the samples and scanning process with Light‐CT system). However, the scanning time could be extended when the machine failed to analyze the samples, as the analysis had to be restarted.

**FIGURE 1:**
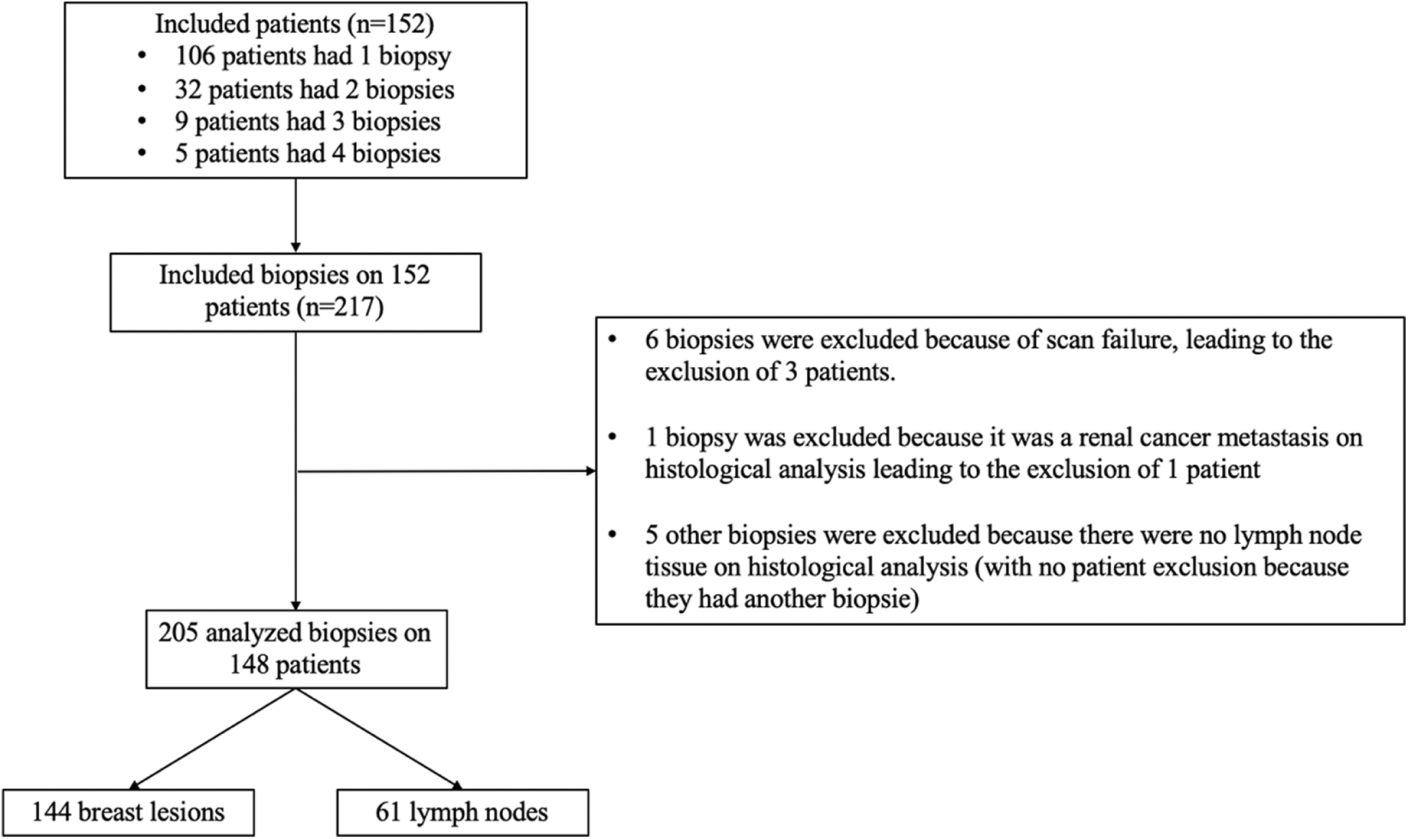
Flow chart.

Some samples could not be analyzed in DCI or FF‐OCT because of total or partial failure. Indeed, procedural failures could occur. Among the 420 samples analyzed in FF‐OCT and DCI, 63 were in complete failures (FF‐OCT and DCI) and 31 in partial failures (FF‐OCT or DCI). When we failed to obtain a FF‐OCT or a DCI image for all the samples of one biopsy, it leaded to its exclusion. These failures led to the exclusion of six biopsies. Six other biopsies were excluded for reasons summarized in flow chart (Figure 1). Those 12 excluded biopsies leaded to the exclusion of 4 patients (who had only one biopsy). Finally, the analysis included 205 biopsies with 144 breast lesions and 61 lymph nodes on 148 patients.

The mean age of the patients was 54.9 years with a mean body mass index (BMI) at 26.1. 54.9% of the patients were menopaused, and among them, 23.1% had a hormonal replacement therapy (HRT). Other characteristics are summarized in the Table 1.

**TABLE 1:** Patient characteristics.

Label	Variable	Value
Age at the inclusion (years)	Min/Max	19/93
	Med [IQR]	54.0 [44.8–65.3]
	Mean (std)	54.9 (15.9)
	*N* (NA)	148 (0)
Age of first menstruation (years)	Min/Max	9/19
	Med [IQR]	13 [12–14]
	Mean (std)	12.8 (1.7)
	*N* (NA)	129 (19)
Age at first child (years)	Min/Max	16/42
	Med [IQR]	26 [23–29]
	Mean (std)	26.4 (5.2)
	*N* (NA)	108 (40)
Menopause	No	*N* (%) = 64 (45.1)
	Yes	*N* (%) = 78 (54.9)
	NA	*N* = 6
HRT	No	*N* (%) = 60 (76.9)
	Yes	*N* (%) = 18 (23.1)
	NA	*N* = 70
Duration of HRT (years)	Min/Max	0.25/20
	Med [IQR]	10 [5–10]
	Mean (std)	8.9 (5.6)
	*N* (NA)	15 (133)
BMI	Min/Max	17.3/93.5
	Med [IQR]	24.5 [22.2–28.5]
	Mean (std)	26.1 (8.2)
	*N* (NA)	143 (5)
Breast density	A–B	*N* (%) = 45 (32.1)
	C–D	*N* (%) = 95 (67.9)
	NA	*N* = 4
First degree family history of breast cancer	No	*N* (%) = 121 (83.5)
	Yes	*N* (%) = 24 (16.5)
	NA	*N* = 3
History of breast surgery or biopsy	No	*N* (%) = 66 (45.5)
	Yes	*N* (%) = 79 (54.5)
	NA	*N* = 3

Abbreviations: BMI, body mass index; HRT, hormonal replacement therapy.

Birads grades were distributed as follows among breast masses: 22 (15.3%) ACR3, 85 (59.0%) ACR4, and 37 (25.7%) ACR5.

On breast tissue, there were 69 invasive cancers (47.9%), 70 benign lesions (48.3%), 3 in situ cancer (2.1%), and 2 atypias (1.4%). There were 32 metastatic nodes (52.5%).

Among invasive cancers, 18 were triple negative (17.8%), 63 were luminal cancer (62.4%), and 18 were HER2+ (17.8%). The mean size of biopsied breast lesions was 17 mm and 19 mm when it was cancerous. The mean size of biopsied lymph nodes was 16 mm.

The prevalence of cancer in the various ACR categories was consistent with the literature on the Birads classification^16^: 0% in ACR3 and 4a, 33.3% in ACR4b, 76.5% in ACR4c, and 94.6% in ACR5 (Table A1).

### 3.2 Dynamic cell imaging and full‐field optical coherence tomography images major characteristics

The normal structures of lymph nodes and breast tissue were easily discernible. In FF‐OCT and DCI, fat tissue was visible as a honeycomb‐like black matrix. As a result, it was possible to easily identify fat‐only samples (mostly for lymph node biopsy), which can be useful in order to identify samples that cannot be interpreted in histology because they did not include the breast lesion or the lymph node of interest. The breast structures (lobules, galactophoric ducts, vessels, and extracellular matrix) were also clearly visible as well‐organized, dark cells in FF‐OCT and yellow/green double cell layers in DCI. This made possible to identify disorganized specimens in which these structures did not appear or were disrupted.

**FIGURE 2:**
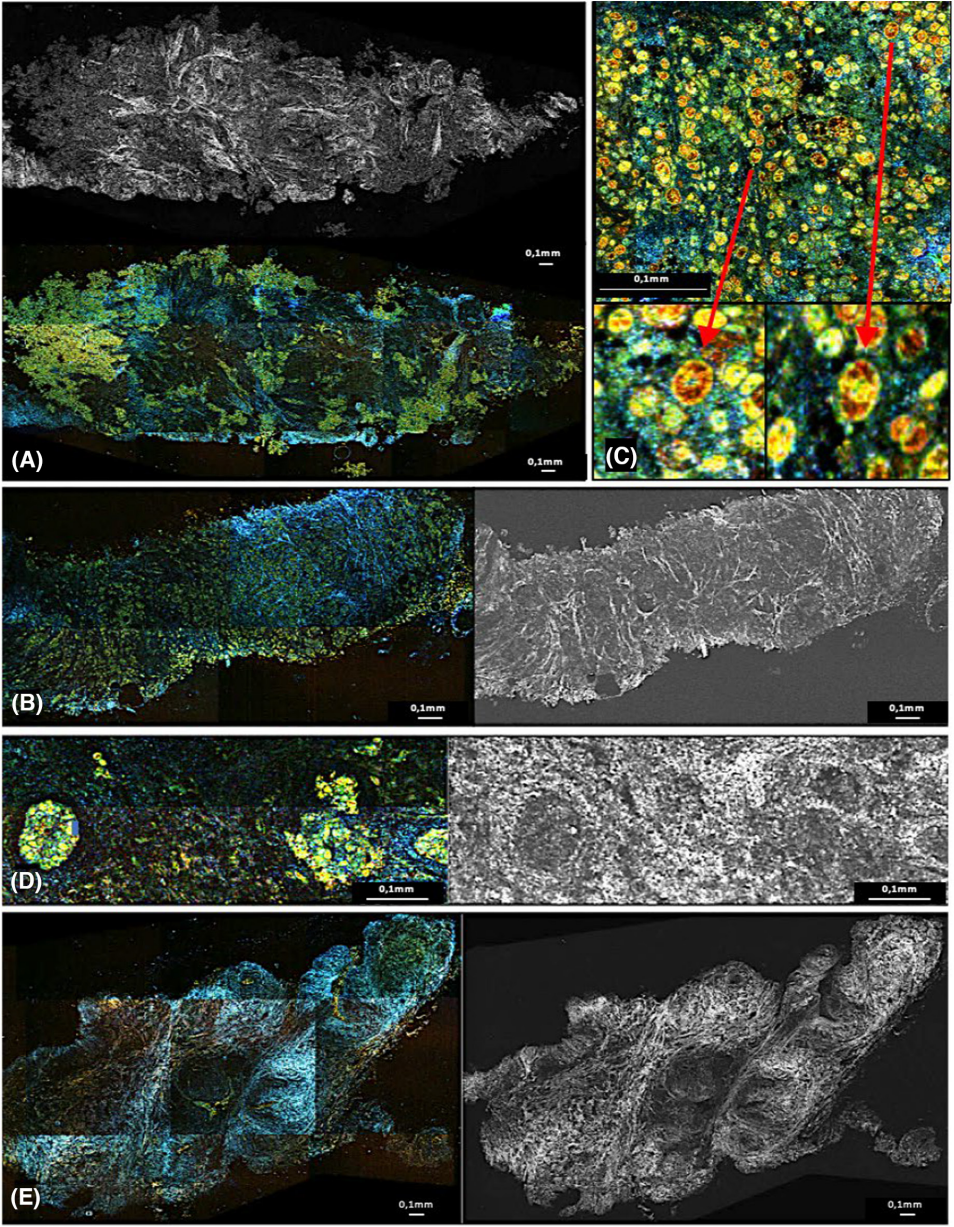
Images in dynamic cell imaging (DCI) and full‐field optical coherence tomography (FFOCT): two infiltrating cancer diagnosed on pathology (A,B). Focusing on cells with ×10 enlargement on bottom images (C). Normal galactophoric duct (D). Adenofibroma (E). In DCI, cells appear as yellow formation with black or red core, extracellular matrix appear as blue linear formations. We see the cellular infiltrate visible in gray/dark on the FFOCT image and in green/yellow on the DCI image. The cells are grouped in clusters or linearly. This is in contrast to the near absence of cells in the adenofibroma or the normal organization of the galactophoric ducts. Irregular and sharps zone of fibrosis visible in bright white (FFOCT) and in blue (DCI) surrounds these infiltrates corresponding to cancerous stroma reaction (A) and is different to the fibrosis visible as gray/white matter visible on the adenofibroma (F) or next to the normal duct (E). Focusing on these infiltrates, we see that the cells are abnormal, irregular in shape and size, and composed of clusters of red/black matter. “Double” cells that may correspond to mitoses are visible. (C) They are opposed to the normal small and similar sizes cells without red clusters and well arranged in two layers within the galactophoric ducts.

During the first readings, each reader noticed that malignant or suspicious breast samples showed high cellularity with regular or non‐regular hypointensities in FF‐OCT corresponding to active infiltrating cells arranged in clusters, in a linear fashion, or occupying the entire sample (Figure 2A–C). On DCI, these cells appeared yellow/green. Compared to non‐cancerous cells, they frequently exhibited atypias with large red and black clumps that may correspond to their nuclear material. Double cells, most likely in mitosis, were visible in few samples. (Figure 2C).

**FIGURE 3:**
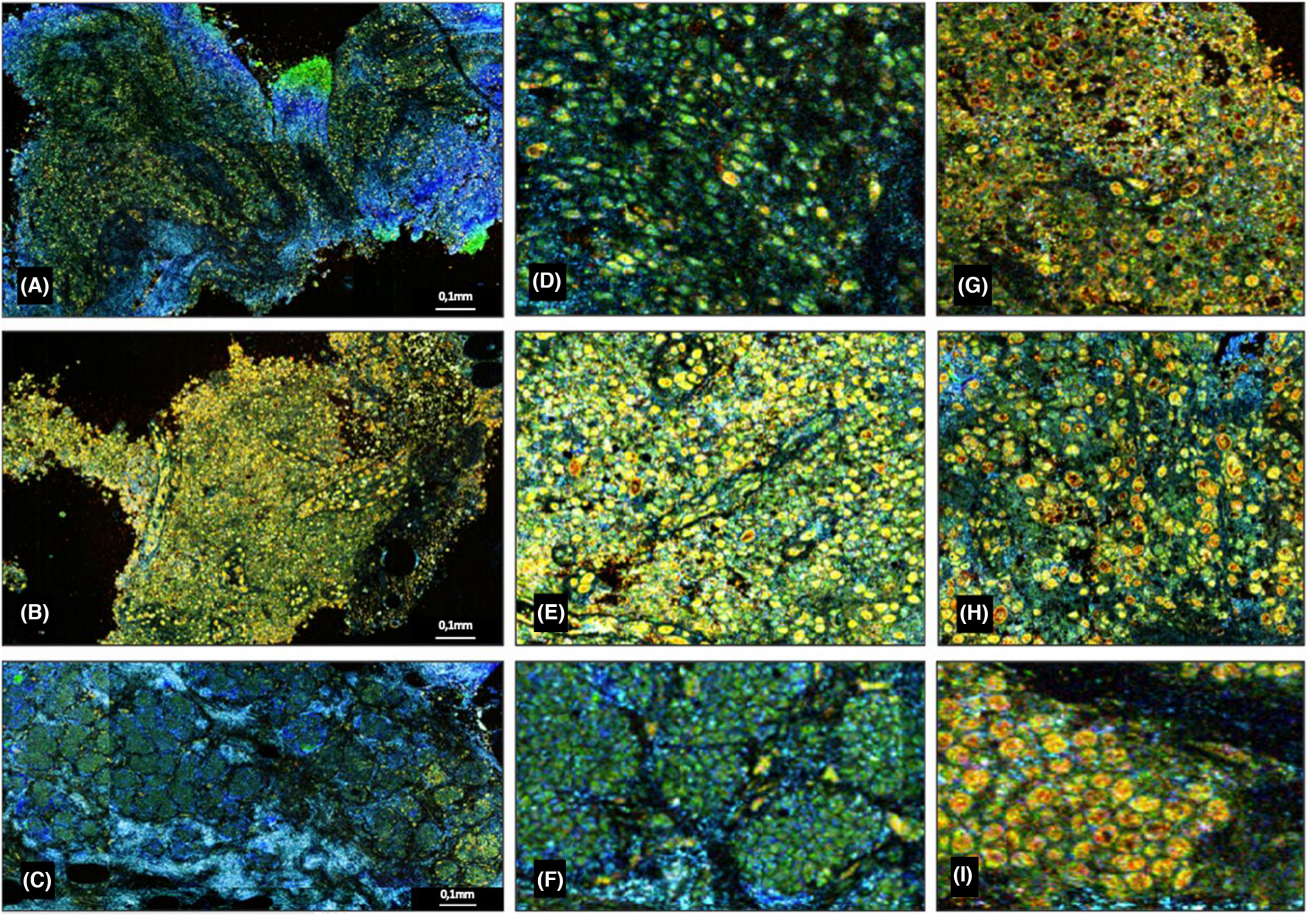
Dynamic cell imaging (DCI) images. Papilloma (A). Histiocytosis (B). Adenoma (C). ×10 focusing on each samples (D–F) with cancerous cell comparison (G–I). In DCI, cells appear as yellow formation with black or red core, extracellular matrix appear as blue linear formations. We can observe that benign specimens can also contain a large cellularity visible as green/yellow cells (A–C). But we notice that they contain more regular and cohesive cells (D–F) with less red/black clumps compared to the malignant specimens (GI). Finally, for adenomas, they are grouped and well‐organized (C).

Following these initial observations, readers identified potential traps, with very cellular samples in FF‐OCT (gray infiltrate) and DCI (green/yellow cells), that may be taken for cancers. The majority of these traps were histiocytosis, papillomas, and adenomas (Figure 3). However, they could be discriminated from cancer because the cells were smaller, more cohesive, and did not contain any black/red clumps (Figure 3B, E, H). Identifying clusters of suspicious cells surrounded by irregular fibrosis with trabeculations visible as blue in DCI and bright white in FF‐OCT was also very helpful to identify carcinologic foci on the samples (Figure 2).

**FIGURE A1:**
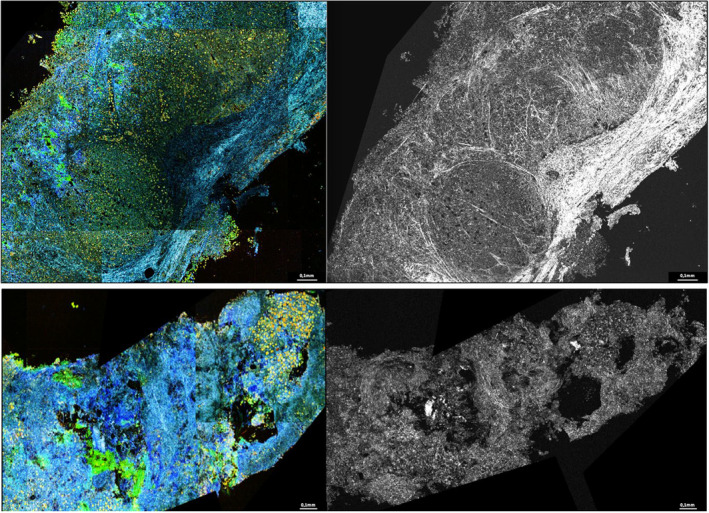
Benign and malignant lymph nodes. In DCI, cells appear as yellow formation with black or red core, extracellular matrix appear as blue linear formations. We can notice a well‐defined lymphatic lobule on this benign lymph node image, the cortex is respected and no irregularity is noted. The cellular organization is regular within the lobule without pathological clusters. In contrast, the second specimen shows a complete disorganization with many irregularities. Clusters of pathological cells can be seen, particularly in the upper right‐hand corner of the image.

The lymph nodes could be identified by the high cellularity of the lymphoid follicles, which were surrounded by a continuous capsule. Frequently, the hilum was visible. (Figure A1).

Structural elements were particularly helpful for lymph nodes because the samples were all very cellular, and it was difficult to identify pathologic cells gathered in metastatic clusters among all the normal lymphoid or inflammatory cells. We could thus identify capsular disturbances, abnormalities or absence of hilum, irregularities of adipocytes next to metastasis, and disruptions of lymphoid follicles. This structural analysis of the samples, which is a key element of the reading, was not immediately apparent to non‐pathologist readers. At the end of every lectors' interpretations when sharing our observations, this diagnosis key element was identified by the pathologist, mostly on nodes.

**FIGURE 4:**
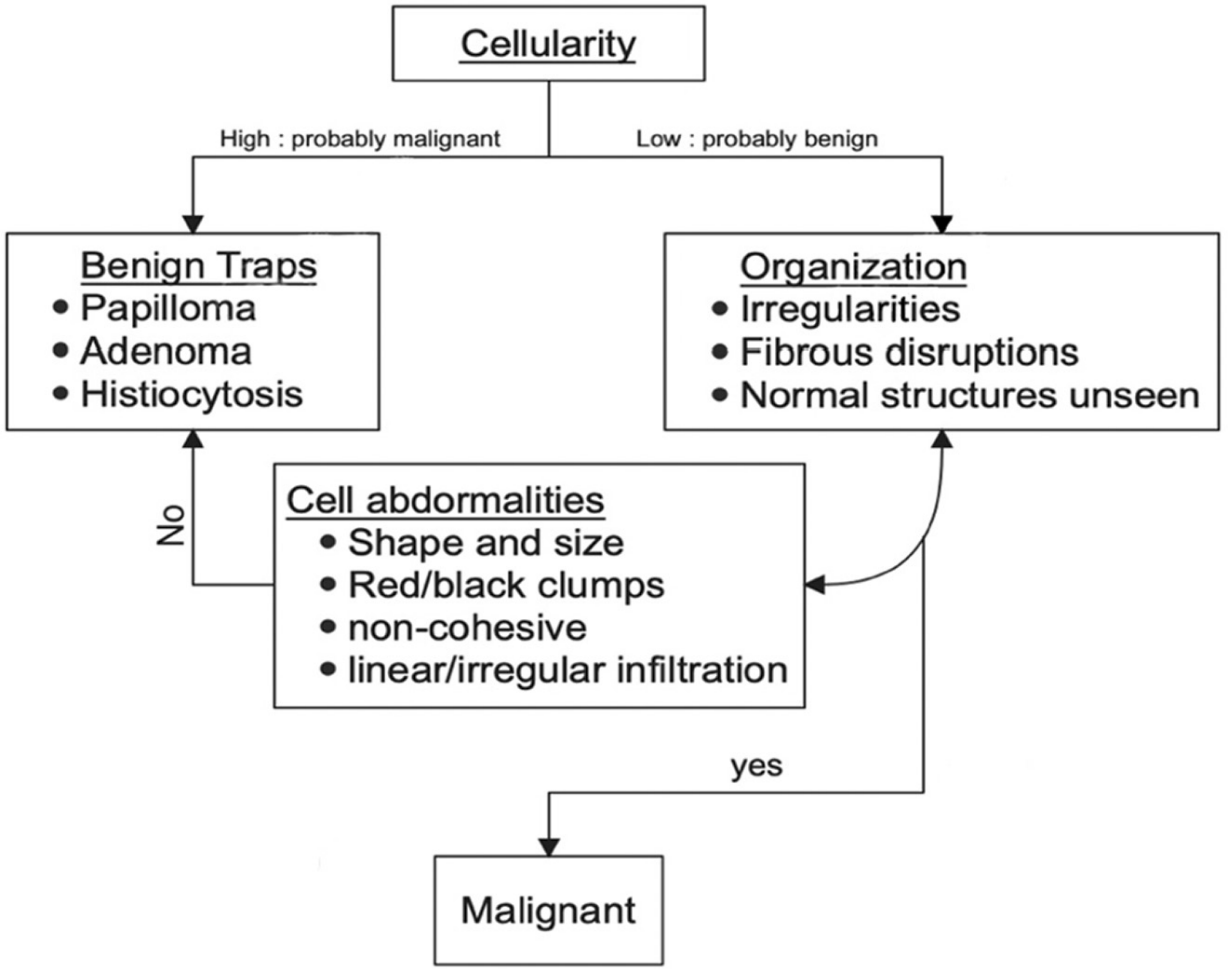
Diagnosis algorithm.

We developed a diagnostic algorithm for D‐FFOCT readings (Figure 4) thanks to the synthesis of all these previous elements: First, we observe the cellularity; samples with numerous cells are typically malignant. For these samples, it is necessary to be aware of the traps that can be identified based on the cellular organization and appearance of the cells. Therefore, it will be simple‐to‐identify histiocytosis, adenomas, and papillomas. Less cellular samples will often be benign lesion; it is important to look at the structural elements (irregularities, fibrous disruption, normal structures present or not) and finally to observe the few cell characteristics in order to identify small carcinogenic spots.

### 3.3 Diagnostic performances

The main objective of the study, i.e., demonstrating a PPV significantly greater than 70% by the senior radiologist, was not reached. Although three of the four readers reached an estimated positive predictive value greater than 70%, the lower bound of the 95% CI was greater than 70% for none of them (Table 2). There was substantial variability on specificity between the readers (from 49% to 70%) as well as on sensitivity (from 69% to 85%). Averaging (GEE model) over the four readers, the results yielded 0.77 [0.7; 0.82] for sensitivity; 0.64 [0.58; 0.71] for specificity; 0.74 [0.68; 0.78] for PPV, and 0.75 [0.72; 0.78] for NPV.

**TABLE 2:** Overall diagnostic performance and for each reader.

	Sensitivity	Specificity	PPV	NPV
Senior radiologist	0.85 [0.76; 0.91]	0.49 [0.39; 0.6]	0.66 [0.57; 0.74]	0.78 [0.66; 0.87]
Junior radiologist	0.73 [0.63; 0.81]	0.7 [0.6; 0.79]	0.78 [0.69; 0.86]	0.81 [0.71; 0.89]
Surgeon	0.78 [0.69; 0.85]	0.69 [0.59; 0.78]	0.74 [0.65; 0.82]	0.75 [0.65; 0.83]
Pathologist	0.69 [0.59; 0.78]	0.69 [0.59; 0.78]	0.77 [0.68; 0.85]	0.76 [0.65; 0.84]
Overall	0.77 [0.7; 0.82]	0.64 [0.58; 0.71]	0.74 [0.68; 0.78]	0.75 [0.72; 0.78]

Specificity was substantially higher for breast tissue than for lymph node biopsies (0.72 vs. 0.46, *p* < 0.001). By taking breast biopsy alone, the results were 0.78 [0.7; 0.84] for sensitivity, 0.72 [0.64; 0.79] for specificity with 0.78 [0.73; 0.82] PPV and 0.78 [0.74; 0.81] NPV.

Regarding lymph nodes, the results were 0.75 [0.61; 0.85] for sensitivity and 0.46 [0.33; 0.6] for specificity. The pathologist had a significantly higher specificity estimate than all other readers, with a 0.69 [0.56; 0.79] specificity.

There were no significant variations of sensitivity according to overall histological type (*p* = 0.26), histological grade (*p* = 0.45), and breast density (*p* = 0.62). However, the specificity was significantly lower for adenomas, papilloma's, and histiocytosis than the other benign lesion (*p* = 0.012).

When combining ACR 3, 4a, and 4b, a sensitivity of 0.5 and a specificity of 0.74 were estimated, resulting in a 90% negative predictive value. For ACR 4c and 5, we obtained a sensitivity of 0.81 and a specificity of 0.57, resulting in a 95% positive predictive value.

The experience of the readers had no statistically significant effect on the results (See Appendix 1), and the readers quickly achieved maximum sensitivity and specificity.

## 4 DISCUSSION

### 4.1 Global results

In this study, considering nodes and breast lesion together, despite a point estimate of 74%, our results do not reach significance in demonstrating a positive predictive value significantly higher than 70% as we planned, neither with the senior radiologist nor when taking into account the 4 readers. The results were the poorest on lymph nodes; indeed the specificity was significantly worse. However, results on breast lesions taken alone are interesting for the use of this technique in daily practice, and we will see why the main results did not meet our expectations. Other studies have already shown good correlation between conventional pathology and FFOCT images. In a previous study by Assayag et al.,^13^ sensitivity and specificity of FF‐OCT detecting breast cancer were roughly 90% and 75%, respectively. In a recent study, Yang and colleagues^17^ used DCI combined with FF‐OCT (D‐FFOCT), similarly to our study, to explore the margin of breast cancer with very good results, with both sensitivity and specificity exceeding 90%.

### 4.2 Breast lesions

In this study, by taking only breast lesions, the results reached our expected values, with 0.78 sensitivity and 0.72 specificity with both 0.78 PPV and NPV. Moreover, the NPV reached 90% for ACR 3, 4a, and 4b and the PPV 95% for ACR 4c and 5. With the use of D‐FF‐OCT in daily practice, it would thus have been possible to reassure a majority of patients with negative images and low ACR referred for breast cancer suspicion. On the opposite, to quickly refer patients with suspect lesions and positive D‐FFOCT images in a breast cancer care pathway.

However, considering all patients without indications on ACR classification the results remain poorer than in the literature on breast lesions. Those results can first be explained by a lack of experience.

Indeed, as the objective of this study was to evaluate diagnosis performance in daily practice with no dedicated formation, the readers were not specifically trained to interpret these images. The specificity and thus the PPV could have been better by increasing the readers' experiences. For example, on benign breast lesions with high cell count (papillomas, histiocytosis, and adenomas which represented 24% of the benign lesions with a significant lower specificity), readers were tempted to interpret as cancers without identifying characteristics that allowed them to be identified as traps. Besides, after the end of the image reading and by combining our observations, all readers understood their mistakes, and a lot of misdiagnoses may have been avoided, but further studies are needed to show it.

### 4.3 Lymph nodes

With lymph nodes taken alone, the results were poorer, and it contributed to decrease our global results. The lack of experience has also been a determining factor here. The results showed that the pathologist had better outcomes than other readers on lymph nodes. Indeed, it was a lot more difficult to interpret node tissue because of structural analysis that was harder for readers with no pathological experience. They were frequently tempted to conclude that lymph nodes were cancerous due to their high cell content, explaining the lack of specificity on lymph nodes. It shows that pathological knowledge is required to interpret lymph node tissue on D‐FFOCT images. Lymph nodes assessment using FF‐OCT with trained pathologists readers showed better results in the literature.^18^, ^19^ The results should have been similar or even better using D‐FFOCT, and further studies are needed with trained readers to show the feasibility of sentinel nodes assessment with D‐FFOCT.

### 4.4 Learning curves

**FIGURE A2:**
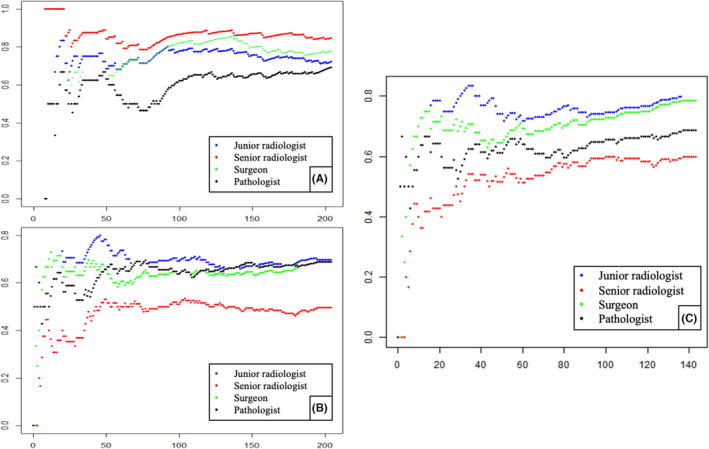
Learning curves. A and B: sensitivity (A) and specifiticity (B) on the *y*‐axis. The number of lectures is on the *x*‐axis. C: concerning only the breast lesions with specificity on *y*‐axis and number of lectures on the *x*‐axis.

We created learning curves based on the readers' experiences during the examination of the 205 biopsies (Figure A2). These curves did not show a significant improvement in diagnostic performance, likely because the strength and the number of readings were insufficient to show it. However, we observed that readers improved rapidly during the first few readings and then quickly reached a plateau depending on the acquired analysis method. By the end of the readings, when the lectors gathered their analysis methods, they noticed that they had adopted different approaches rapidly during the initial readings, but did not modify it or improve them significantly thereafter. This explains the initial improvement and then the plateau reached.

By taking only breast lesions, we observed a tendency, with improvement in the specificity during the readings (Figure 2C). This tendency can be explained by the ability to better detect traps on benign lesion. It shows the importance of experience in the analysis of D‐FFOCT images. With trained lectors and the use of standardized methodology to analyze, these images the results should be better.

### 4.5 Technical feasibility

Another limitation of this study was the feasibility of the technique. The depth of D‐FFOCT slices was determined arbitrarily, and the acquisition of a single depth level on one sample could require up to 10 min on DCI. Furthermore, the time required could be extended when the machine failed to scan samples as the process had to be relaunched. Occurrence of failure was not negligible since out of 420 scanned samples, 63 were in total failure (DCI and FFOCT) and 31 in partial failure (DCI or FFOCT) leading to the exclusion of six biopsied lesion.

For one biopsy with multiple samples, it was not technically possible within the radiologist organization to image several depth levels, multiplying images to scan and therefore the time required. This resulted in a lower sensitivity, particularly for nodes, as small carcinologic foci could be missed on other depth levels, which were found on standard histology with multiple levels of slice. With better organization and faster D‐FFOCT acquisition, multiple depth levels could be imaged thus improving performances on freshly taken biopsies.

Also, most other studies using FF‐OCT technology in oncology have used surgical specimens instead of biopsies. Using fresh biopsy may have contributed to decrease performances compared to other studies. In Yang et al. study, images were obtained from surgical specimens of benign and malignant breasts lesions, resulting in high‐quality samples. Pathologists could analyze tissue with high concordance between conventional pathology and D‐FFOCT images with same level of slides and chosen specimens. They trained two surgeons to read the same samples, and the results were consequently positive. It showed very good concordances between classic pathology and D‐FFOCT images under optimal conditions, pathologists comparing exactly the same slides analyzed in D‐FFOCT and in standard pathology. The diagnostic performances on freshly taken biopsies were often inferior with sensitivity and specificity values closer to our study.^20^

Nonetheless, as explained earlier, fat‐only samples could be identified with FFOCT images as black honeycomb matrix. Therefore, this technique which can be carried out relatively quickly, directly in the radiologist's cabinet, could also be a useful tool to check the quality of the samples before sending them to standard pathology. Indeed, in daily practice, pathologists can fail to interpret biopsies because of poor‐quality sampling, leading to long additional delays.

### 4.6 Other results

Regardless of statistical results, this study also helped to collect a large atlas of D‐FFOCT breast cancer and node images, and by combining the reader's observations, it has been possible to develop a diagnostic algorithm. This diagnostic algorithm, as well as the image atlas, may be used in future studies to train readers to improve diagnosis performances.

It would also be beneficial to use artificial intelligence to develop automatic reading. Indeed, artificial intelligence algorithms have already showed great performances in detecting cancer on breast samples^21^ and one study on basal cell carcinoma showed good diagnostic performances with D‐FFOCT technology using machine learning.^22^ The next step will be to use the images and data collected to perform a study with machine learning on this technique.

## 5 CONCLUSION

This study showed convincing diagnosis performances detecting breast cancer on freshly taken biopsies with D‐FFOCT. It showed that the technique was achievable in daily practice for patients undergoing breast biopsies and also gives radiologists a great tool to check the quality of their samples before sending them to traditional pathology in order to avoid long additional delays. However, the results could be better, mostly on nodes, and did not reach our expectations and show that the technique is not ready to be used in clinical practice. It is mostly explained by the lack of experience of the readers and further studies with trained radiologists. The use of deep learning is another promising field of research. It is essential to keep in mind that this technique cannot replace traditional pathology which is necessary for immunochemistry and molecular analysis, but the two techniques are not mutually exclusive and can be performed on the same sample.

## AUTHOR CONTRIBUTIONS

**Alexis Simon:** Formal analysis (equal); methodology (equal); writing – original draft (equal); writing – review and editing (equal). **Yasmina Badachi:** Conceptualization (equal); formal analysis (equal); writing – review and editing (equal). **Jacques Ropers:** Data curation (equal); investigation (equal); methodology (equal); supervision (equal); writing – review and editing (equal). **Isaura Laurent:** Formal analysis (equal); methodology (equal). **Lida Dong:** Formal analysis (equal); writing – review and editing (equal). **Elisabeth Da Maia:** Formal analysis (equal); writing – review and editing (equal). **Agnès Bourcier:** Data curation (equal); formal analysis (equal). **Geoffroy Canlorbe:** Formal analysis (equal); writing – original draft (equal); writing – review and editing (equal). **Catherine Uzan:** Conceptualization (equal); formal analysis (equal); investigation (equal); methodology (equal); writing – original draft (equal); writing – review and editing (equal).

## FUNDING INFORMATION

The performed study whose methods and results are detailed hereby was partially financed by LLTech Management (equipment on free loan, staff for data management). Data Analysis, statistics, radiological technics were performed without funding. Address: LLTech Management, 58 rue du Dessous des Berges – 75013 Paris, France).

## CONFLICT OF INTEREST STATEMENT

The authors made no disclosures.

## ETHICS APPROVAL AND PATIENT CONSENT STATEMENT

In accordance with French law, all patients were informed about the study objectives and methods and did not oppose to the use of their personal data and biopsy samples. On February 4, 2020, the Institutional Review Board, (IRB00003888, Inserm Ethics Evaluation Committee CEEI) approved this study.

## CLINICAL TRIAL REGISTRATION

The study was registered in ClinicalTrials.gov on March 3, 2020, under the identifier NCT04292821.

## STATEMENT

This study aimed to assess the diagnostic performance of full‐field optical coherence tomography combined with dynamic cell imaging (D‐FFOCT) for breast cancer diagnosis, offering a nondestructive technique that closely approximates histopathological analysis with sufficient spatial resolution. Results showed sensitivity of 77%, specificity of 64%, and highlighted the promising potential for trained radiologists or using machine learning in quick detection of breast cancer.

## Data Availability

The data that support the findings of this study are available from the corresponding author upon reasonable request.

## APPENDIX 1

**TABLE A1:** Final diagnosis according to the Birads classification.

Label	Variable	Atypias	Benign	CIS	Invasive cancer
ACR	3	0 (0%)	22 (100%)	0 (0%)	0 (0%)
	4a	1 (3.7%)	26 (96.3%)	0 (0%)	0 (0%)
	4b	1 (4.17%)	15 (62.5%)	0 (0%)	8 (33.33%)
	4c	0 (0%)	6 (17.65%)	2 (5.88%)	26 (76.47%)
	5	0 (0%)	1 (2.7%)	1 (2.7%)	35 (94.59%)

Abbreviations: ACR, American college of radiology (Birads classification); CIS, Carcinoma in situ.
